# 2,2′-(Piperazine-1,4-di­yl)diethanaminium dibenzoate

**DOI:** 10.1107/S1600536812030115

**Published:** 2012-07-07

**Authors:** Ignacy Cukrowski, Adedapo S. Adeyinka, David C. Liles

**Affiliations:** a Department of Chemistry, University of Pretoria, Private Bag X20, Hatfield 0028, South Africa

## Abstract

The asymmetric unit of the title salt C_8_H_22_N_4_^2+^·2C_7_H_5_O_2_^−^, comprises two independent pairs of half a 2,2′-(piperazine-1,4-di­yl)diethanaminium dication plus a benzoate anion. The dications are symmetrical and lie across crystallographic centres of inversion. The crystal structure was refined as a two-component pseudo-merohedral twin using the twin law 001 0-10 100 [he domain fractions are 0.8645 (8) and 0.1355 (8)]. The anions and cations are linked by N—H⋯O hydrogen bonds and weak N—H⋯O inter­molecular inter­actions to form infinite two-dimensional networks parallel to [101]. The conformation adopted by the cation in the crystal structure is very similar to that adopted by the same cation in the structures of the 2-hy­droxy­benzoate [Cukrowski *et al.* (2012 ▶). *Acta Cryst*, E**68**, o2387], the nitrate and the tetra­hydrogen penta­borate salts.

## Related literature

For the structures of the 2-hy­droxy­benzoate, the nitrate and the tetra­hydrogen penta­borate salts of the 1,4-di(2-ammonio­eth­yl)piperazine cation, see: Cukrowski *et al.* (2012 ▶); Junk & Smith (2005 ▶); Jiang *et al.* (2009 ▶), respectively.
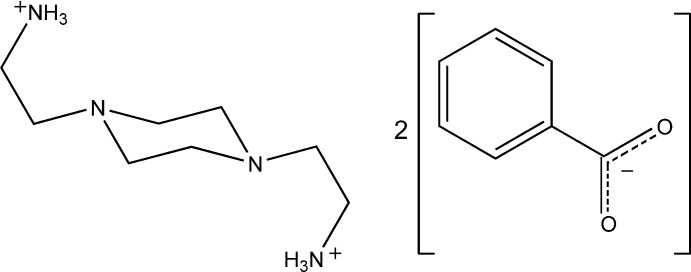


## Experimental

### 

#### Crystal data

C_8_H_22_N_4_^2+^·2C_7_H_5_O_2_^−^
*M* *_r_* = 416.52
Monoclinic, 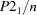

*a* = 19.5300 (4) Å
*b* = 6.6694 (2) Å
*c* = 19.6178 (4) Å
β = 115.989 (1)°
*V* = 2296.89 (10) Å^3^
*Z* = 4
Mo *K*α radiation
μ = 0.08 mm^−1^
*T* = 180 K
0.28 × 0.23 × 0.12 mm

#### Data collection

Nonius KappaCCD diffractometer
Absorption correction: multi-scan (*SORTAV*; Blessing, 1995 ▶) *T*_min_ = 0.910, *T*_max_ = 0.991
20137 measured reflections
5194 independent reflections
3970 reflections with *I* > 2σ(*I*)
*R*_int_ = 0.031

#### Refinement

*R*[*F* ^2^ > 2σ(*F* ^2^)] = 0.040
*wR*(*F* ^2^) = 0.110
*S* = 1.02
5194 reflections
290 parameters
H atoms treated by a mixture of independent and constrained refinement
Δρ_max_ = 0.16 e Å^−3^
Δρ_min_ = −0.18 e Å^−3^

### 

Data collection: *COLLECT* (Nonius, 1998 ▶); cell refinement: *SCALEPACK* (Otwinowski & Minor, 1997 ▶); data reduction: *DENZO* (Otwinowski & Minor, 1997 ▶), *SCALEPACK* and *SORTAV* (Blessing, 1995 ▶); program(s) used to solve structure: *SIR92* (Altomare *et al.*, 1994 ▶); program(s) used to refine structure: *SHELXL97* (Sheldrick, 2008 ▶); molecular graphics: *ORTEP-3 for Windows* (Farrugia, 1997 ▶), *POV-RAY* (Cason, 2004 ▶) and *Mercury* (Macrae *et al.*, 2008 ▶); software used to prepare material for publication: *SHELXL97* and *PLATON* (Spek, 2009 ▶).

## Supplementary Material

Crystal structure: contains datablock(s) I, global. DOI: 10.1107/S1600536812030115/jj2134sup1.cif

Structure factors: contains datablock(s) I. DOI: 10.1107/S1600536812030115/jj2134Isup2.hkl

Supplementary material file. DOI: 10.1107/S1600536812030115/jj2134Isup3.cml

Additional supplementary materials:  crystallographic information; 3D view; checkCIF report

## Figures and Tables

**Table 1:** Hydrogen-bond geometry (Å, °)

*D*—H⋯*A*	*D*—H	H⋯*A*	*D*⋯*A*	*D*—H⋯*A*
N1—H1*A*⋯O15^i^	0.945 (18)	1.833 (19)	2.7739 (17)	173.0 (15)
N1—H1*B*⋯O15	0.902 (16)	1.895 (17)	2.7836 (15)	167.8 (14)
N1—H1*B*⋯O14	0.902 (16)	2.632 (16)	3.2580 (16)	127.2 (13)
N1—H1*C*⋯O14^ii^	0.925 (18)	1.887 (18)	2.7660 (16)	157.9 (14)
N1′—H1′*A*⋯O14′	0.882 (19)	1.857 (19)	2.7355 (17)	174.2 (15)
N1′—H1′*B*⋯O14′^iii^	0.897 (17)	1.908 (17)	2.7836 (16)	164.8 (15)
N1′—H1′*B*⋯O15′^iii^	0.897 (17)	2.533 (16)	3.1858 (16)	130.1 (13)
N1′—H1′*C*⋯O15′^iv^	0.916 (18)	1.934 (18)	2.7585 (16)	148.8 (14)

Symmetry codes: (i) 
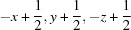
; (ii) 
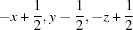
; (iii) 
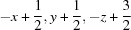
; (iv) 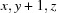.

**Fig. 1.**
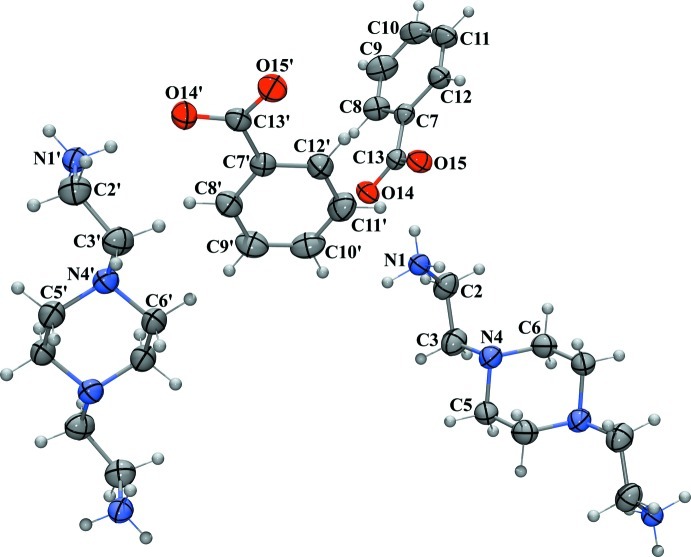
Molecular structure of the title compound showing the atom labeling scheme and 50° probability displacement ellipsoids.

**Fig. 2.**
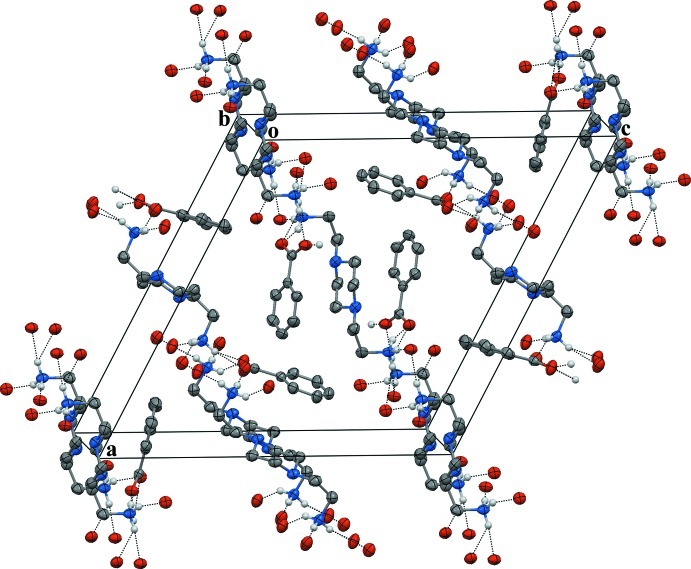
Packing diagram of the title compound viewed offset from along the b axis. Dashed lines indicate N—H···O and O—H···O hydrogen bonds. The intermolecular N—H···O hydrogen bonds form a two-dimensional network.

## supplementary crystallographic information

### Comment

The title compound [C_8_H_22_N_4_^2+^ 2(C_7_H_5_O_2_^-^)] (1) was obtained as an unintended product during an attempt to prepare a benzoate salt of a singly protonated *N*,*N*'-di(2-aminoethyl)-2-aminoethane-1-ammonium ion (C_6_H_19_N_4_^+^ C_7_H_5_O_2_^-^). This occurred because the starting material, instead of being pure *N*,*N*'-di(2-aminoethyl)-ethane-1,2-diamine, (C_6_H_18_N_4_), was a mixture of that compound and 1,4-di(2-aminoethyl)piperazine (C_8_H_22_N_4_) see Cukrowski, *et al.* (2012).

The asymmetric unit of the title compound, C_8_H_22_N_4_^2+^, 2(C_7_H_5_O_2_^-^), 1, is a salt with two independent pairs of half a C_8_H_22_N_4_^2+^ cation plus a C_7_H_5_O_2_^-^ anion. The C_8_H_22_N_4_^2+^ cations are symmetrical and lie across crystallographic centres of inversion (Fig. 1). The crystal structure was refined as a two-component pseudo- merohedral twin using the twin law 0 0 1 0 - 1 0 1 0 0. The fractional contribution refined to 0.1355 (8).

All three H atoms of each ammonium group in the cations of 1 are hydrogen bonded to the O atoms of the carboxylate groups of the anions. For each ammonium group, one H atom forms a bifurcated hydrogen bond to both of the O atoms of the carboxylate group of one anion, whereas the other two H atoms each form single hydrogen bonds to one O atom of the carboxylate group of each of two additional anions (Fig. 2). Thus both the O atoms of each carboxylate group are each acceptors for two hydrogen bonds. N—H···O hydrogen bonds and weak N—H···O intermolecular interactions link the cations and anions to form a two-dimensional network with layers parallel to the [101] plane (Fig. 2). Each of the two independent cation-anion pairs form the content of alternate network layers.

The conformation adopted by the C_8_H_22_N_4_^2+^ cation in the crystal structure of 1 is very similar to the conformations adopted by the same cation in the crystal structures of the 2-hydroxybenzoate (Cukrowski, *et al.*, 2012), the nitrate (Junk & Smith, 2005) and the tetrahydrogenpentaborate (Jiang, *et al.*, 2009) salts despite the differences in the size and shape of the anions in the various structures.

### Experimental

2 ml of a.3.32 *M* aqueous solution of what was claimed by the supplier (QinHuangDao JinLei Chemical Co.Ltd) to be *N*,*N*'-di(2-aminoethyl)-ethane-1,2-diamine, but which turned out to be a mixture of that compound (C_6_H_18_N_4_, 6.64*n* mmol) and 1,4-di(2-aminoethyl)piperazine (C_8_H_20_N_4_, 5.57(1-*n*) mmol) was added to 0.78 g of benzoic acid (6.96 mmol), resulting in a clear colourless solution. 0.2 ml of ethanol was added to the solution and the mixture was heated for 3 h at 70 °C. The solution was cooled to room temperature and then left covered for six days and then allowed to slowly evaporate by covering the container with perforated aluminium foil. Yellow crystals were obtained after four days of slow evaporation.

### Refinement

The crystal structure was refined as a two component pseudo-merohedral twin using the twin law 0 0 1 0 - 1 0 1 0 0. The fractional contribution refined to 0.1355 (8). H1A, H1B and H1C were located by a difference map and their coordinates were refined. All of the remaining H atoms were placed in their calculated positions and then refined using the riding model with Atom—H lengths of 0.95Å, (CH) or 0.99Å (CH_2_). Isotropic displacement parameters for all hydrogen atoms were set to 1.20 times *U*_eq_ of the parent atom.

### Crystal data

**Table:**

C_8_H_22_N_4_^2+^·2C_7_H_5_O_2_^−^	*F*(000) = 896
*M**_r_* = 416.52	*D*_x_ = 1.204 Mg m^−^^3^
Monoclinic, *P*2_1_/*n*	Mo *K*α radiation, λ = 0.71070 Å
Hall symbol: -P 2yn	Cell parameters from 12295 reflections
*a* = 19.5300 (4) Å	θ = 1.0–27.5°
*b* = 6.6694 (2) Å	µ = 0.08 mm^−^^1^
*c* = 19.6178 (4) Å	*T* = 180 K
β = 115.989 (1)°	Block, yellow
*V* = 2296.89 (10) Å^3^	0.28 × 0.23 × 0.12 mm
*Z* = 4	

### Data collection

**Table:**

Nonius KappaCCD diffractometer	5194 independent reflections
Radiation source: fine-focus sealed tube	3970 reflections with *I* > 2σ(*I*)
Graphite monochromator	*R*_int_ = 0.031
Thin slice ω and φ scans	θ_max_ = 27.5°, θ_min_ = 3.6°
Absorption correction: multi-scan (*SORTAV*; Blessing, 1995)	*h* = −25→22
*T*_min_ = 0.910, *T*_max_ = 0.991	*k* = −7→8
20137 measured reflections	*l* = −25→25

### Refinement

**Table:**

Refinement on *F*^2^	Primary atom site location: structure-invariant direct methods
Least-squares matrix: full	Secondary atom site location: difference Fourier map
*R*[*F*^2^ > 2σ(*F*^2^)] = 0.040	Hydrogen site location: difference Fourier map
*wR*(*F*^2^) = 0.110	H atoms treated by a mixture of independent and constrained refinement
*S* = 1.02	*w* = 1/[σ^2^(*F*_o_^2^) + (0.0702*P*)^2^] where *P* = (*F*_o_^2^ + 2*F*_c_^2^)/3
5194 reflections	(Δ/σ)_max_ = 0.001
290 parameters	Δρ_max_ = 0.16 e Å^−^^3^
0 restraints	Δρ_min_ = −0.18 e Å^−^^3^

### Special details

**Table:**

Geometry. All e.s.d.'s (except the e.s.d. in the dihedral angle between two l.s. planes) are estimated using the full covariance matrix. The cell e.s.d.'s are taken into account individually in the estimation of e.s.d.'s in distances, angles and torsion angles; correlations between e.s.d.'s in cell parameters are only used when they are defined by crystal symmetry. An approximate (isotropic) treatment of cell e.s.d.'s is used for estimating e.s.d.'s involving l.s. planes.
Refinement. Refinement of *F*^2^ against ALL reflections. The weighted *R*-factor *wR* and goodness of fit *S* are based on *F*^2^, conventional *R*-factors *R* are based on *F*, with *F* set to zero for negative *F*^2^. The threshold expression of *F*^2^ > σ(*F*^2^) is used only for calculating *R*-factors(gt) *etc*. and is not relevant to the choice of reflections for refinement. *R*-factors based on *F*^2^ are statistically about twice as large as those based on *F*, and *R*- factors based on ALL data will be even larger.

### Fractional atomic coordinates and isotropic or equivalent isotropic displacement parameters (Å^2^)

**Table:**

	*x*	*y*	*z*	*U*_iso_*/*U*_eq_	
N1	0.19472 (7)	0.2447 (2)	0.19552 (7)	0.0375 (3)	
H1A	0.1776 (9)	0.374 (3)	0.2008 (8)	0.045*	
H1B	0.2431 (9)	0.223 (2)	0.2302 (9)	0.045*	
H1C	0.1652 (9)	0.142 (3)	0.2002 (8)	0.045*	
C2	0.19529 (8)	0.2437 (3)	0.12037 (8)	0.0491 (4)	
H2A	0.2127	0.1110	0.1116	0.059*	
H2B	0.2319	0.3457	0.1198	0.059*	
C3	0.11811 (9)	0.2872 (3)	0.05773 (8)	0.0512 (4)	
H3A	0.0994	0.4156	0.0686	0.061*	
H3B	0.1226	0.3034	0.0097	0.061*	
N4	0.06208 (7)	0.12951 (18)	0.04783 (6)	0.0418 (3)	
C5	−0.01461 (8)	0.2057 (2)	0.00103 (8)	0.0458 (4)	
H5A	−0.0182	0.2489	−0.0487	0.055*	
H5B	−0.0242	0.3241	0.0260	0.055*	
C6	0.07429 (8)	−0.0487 (2)	0.01107 (8)	0.0483 (4)	
H6A	0.1254	−0.1048	0.0430	0.058*	
H6B	0.0726	−0.0106	−0.0384	0.058*	
C7	0.46941 (7)	0.2132 (2)	0.30262 (7)	0.0347 (3)	
C8	0.51949 (8)	0.3595 (3)	0.30237 (8)	0.0470 (4)	
H8	0.5013	0.4909	0.2852	0.056*	
C9	0.59622 (9)	0.3155 (3)	0.32714 (9)	0.0583 (5)	
H9	0.6304	0.4175	0.3276	0.070*	
C10	0.62302 (9)	0.1248 (3)	0.35110 (9)	0.0566 (5)	
H10	0.6755	0.0952	0.3680	0.068*	
C11	0.57335 (9)	−0.0231 (3)	0.35052 (8)	0.0512 (4)	
H11	0.5915	−0.1552	0.3664	0.061*	
C12	0.49691 (8)	0.0210 (2)	0.32676 (8)	0.0415 (3)	
H12	0.4631	−0.0809	0.3270	0.050*	
C13	0.38687 (7)	0.2669 (2)	0.27915 (7)	0.0340 (3)	
O14	0.36433 (5)	0.43634 (15)	0.25215 (5)	0.0429 (2)	
O15	0.34467 (5)	0.13526 (15)	0.28820 (6)	0.0435 (3)	
N1'	0.18677 (7)	0.8130 (2)	0.69616 (7)	0.0387 (3)	
H1'A	0.2019 (9)	0.704 (3)	0.6812 (9)	0.046*	
H1'B	0.2193 (9)	0.845 (2)	0.7439 (10)	0.046*	
H1'C	0.1828 (9)	0.917 (3)	0.6640 (9)	0.046*	
C2'	0.11280 (9)	0.7645 (3)	0.69548 (8)	0.0490 (4)	
H2'A	0.0946	0.8814	0.7141	0.059*	
H2'B	0.1193	0.6506	0.7301	0.059*	
C3'	0.05450 (8)	0.7108 (3)	0.61682 (8)	0.0484 (4)	
H3'A	0.0703	0.5854	0.6006	0.058*	
H3'B	0.0049	0.6857	0.6177	0.058*	
N4'	0.04519 (6)	0.86872 (18)	0.56193 (6)	0.0409 (3)	
C5'	0.00761 (8)	1.0480 (2)	0.57298 (8)	0.0458 (4)	
H5'A	0.0379	1.1020	0.6246	0.055*	
H5'B	−0.0434	1.0114	0.5683	0.055*	
C6'	0.00043 (8)	0.7944 (2)	0.48472 (8)	0.0441 (4)	
H6'A	−0.0507	0.7539	0.4785	0.053*	
H6'B	0.0256	0.6747	0.4762	0.053*	
C7'	0.19900 (7)	0.3513 (2)	0.53125 (7)	0.0351 (3)	
C8'	0.17823 (8)	0.5420 (2)	0.50088 (8)	0.0425 (3)	
H8'	0.1815	0.6515	0.5332	0.051*	
C9'	0.15272 (9)	0.5741 (3)	0.42383 (9)	0.0515 (4)	
H9'	0.1377	0.7044	0.4032	0.062*	
C10'	0.14927 (9)	0.4151 (3)	0.37717 (9)	0.0533 (4)	
H10'	0.1315	0.4365	0.3243	0.064*	
C11'	0.17134 (9)	0.2262 (3)	0.40677 (8)	0.0518 (4)	
H11'	0.1699	0.1181	0.3746	0.062*	
C12'	0.19571 (8)	0.1943 (2)	0.48389 (8)	0.0440 (3)	
H12'	0.2102	0.0634	0.5043	0.053*	
C13'	0.22236 (7)	0.3122 (2)	0.61444 (7)	0.0355 (3)	
O14'	0.23245 (6)	0.46100 (17)	0.65735 (5)	0.0521 (3)	
O15'	0.23097 (6)	0.13489 (16)	0.63648 (6)	0.0478 (3)	

### Atomic displacement parameters (Å^2^)

**Table:**

	*U*^11^	*U*^22^	*U*^33^	*U*^12^	*U*^13^	*U*^23^
N1	0.0311 (6)	0.0325 (7)	0.0436 (6)	−0.0018 (5)	0.0115 (5)	−0.0006 (5)
C2	0.0417 (8)	0.0570 (10)	0.0495 (8)	−0.0097 (7)	0.0207 (6)	−0.0086 (7)
C3	0.0576 (9)	0.0497 (10)	0.0432 (8)	−0.0072 (8)	0.0193 (7)	0.0004 (7)
N4	0.0427 (7)	0.0410 (7)	0.0360 (6)	−0.0020 (5)	0.0119 (5)	−0.0013 (5)
C5	0.0519 (9)	0.0402 (8)	0.0360 (7)	0.0059 (7)	0.0107 (6)	−0.0001 (6)
C6	0.0448 (8)	0.0519 (10)	0.0428 (7)	0.0073 (7)	0.0144 (6)	−0.0012 (7)
C7	0.0362 (7)	0.0386 (8)	0.0291 (6)	0.0007 (6)	0.0141 (5)	−0.0019 (5)
C8	0.0428 (8)	0.0523 (10)	0.0458 (8)	−0.0024 (7)	0.0195 (6)	0.0051 (7)
C9	0.0424 (9)	0.0790 (14)	0.0564 (9)	−0.0083 (9)	0.0242 (7)	0.0073 (8)
C10	0.0378 (8)	0.0872 (14)	0.0471 (8)	0.0120 (9)	0.0209 (6)	0.0019 (8)
C11	0.0468 (8)	0.0586 (11)	0.0473 (8)	0.0164 (8)	0.0198 (6)	0.0009 (7)
C12	0.0405 (8)	0.0430 (9)	0.0401 (7)	0.0045 (6)	0.0169 (6)	−0.0012 (6)
C13	0.0375 (7)	0.0331 (8)	0.0299 (6)	0.0004 (6)	0.0133 (5)	−0.0028 (5)
O14	0.0447 (5)	0.0350 (6)	0.0472 (5)	0.0067 (4)	0.0184 (4)	0.0049 (4)
O15	0.0346 (5)	0.0389 (6)	0.0540 (6)	−0.0003 (4)	0.0167 (4)	0.0055 (4)
N1'	0.0444 (7)	0.0342 (7)	0.0334 (6)	−0.0010 (5)	0.0132 (5)	0.0019 (5)
C2'	0.0499 (9)	0.0546 (10)	0.0447 (8)	−0.0003 (7)	0.0227 (7)	0.0058 (7)
C3'	0.0402 (8)	0.0472 (10)	0.0529 (9)	−0.0060 (7)	0.0159 (6)	0.0023 (7)
N4'	0.0353 (6)	0.0389 (7)	0.0423 (6)	−0.0011 (5)	0.0113 (5)	−0.0021 (5)
C5'	0.0419 (8)	0.0485 (9)	0.0432 (8)	−0.0010 (7)	0.0152 (6)	−0.0082 (7)
C6'	0.0376 (7)	0.0406 (9)	0.0476 (8)	−0.0017 (6)	0.0127 (6)	−0.0088 (6)
C7'	0.0308 (6)	0.0379 (8)	0.0375 (7)	−0.0013 (6)	0.0159 (5)	0.0026 (6)
C8'	0.0444 (8)	0.0390 (9)	0.0466 (8)	0.0006 (6)	0.0223 (6)	0.0053 (6)
C9'	0.0560 (9)	0.0530 (10)	0.0492 (8)	0.0069 (8)	0.0265 (7)	0.0185 (8)
C10'	0.0478 (8)	0.0758 (13)	0.0375 (7)	0.0002 (8)	0.0198 (6)	0.0123 (8)
C11'	0.0554 (9)	0.0616 (11)	0.0402 (8)	−0.0002 (8)	0.0226 (7)	−0.0054 (7)
C12'	0.0472 (8)	0.0404 (8)	0.0425 (8)	0.0024 (7)	0.0179 (6)	0.0008 (6)
C13'	0.0312 (7)	0.0365 (8)	0.0388 (7)	0.0026 (6)	0.0154 (5)	0.0036 (6)
O14'	0.0690 (7)	0.0428 (7)	0.0385 (5)	0.0114 (5)	0.0181 (5)	−0.0018 (5)
O15'	0.0582 (7)	0.0390 (6)	0.0432 (5)	−0.0003 (5)	0.0195 (5)	0.0086 (4)

### Geometric parameters (Å, º)

**Table:**

N1—C2	1.4792 (19)	N1'—C2'	1.475 (2)
N1—H1A	0.945 (18)	N1'—H1'A	0.882 (19)
N1—H1B	0.902 (16)	N1'—H1'B	0.897 (17)
N1—H1C	0.925 (18)	N1'—H1'C	0.916 (18)
C2—C3	1.498 (2)	C2'—C3'	1.505 (2)
C2—H2A	0.9900	C2'—H2'A	0.9900
C2—H2B	0.9900	C2'—H2'B	0.9900
C3—N4	1.468 (2)	C3'—N4'	1.460 (2)
C3—H3A	0.9900	C3'—H3'A	0.9900
C3—H3B	0.9900	C3'—H3'B	0.9900
N4—C5	1.4625 (19)	N4'—C6'	1.4640 (18)
N4—C6	1.463 (2)	N4'—C5'	1.468 (2)
C5—C6^i^	1.506 (2)	C5'—C6'^ii^	1.502 (2)
C5—H5A	0.9900	C5'—H5'A	0.9900
C5—H5B	0.9900	C5'—H5'B	0.9900
C6—C5^i^	1.506 (2)	C6'—C5'^ii^	1.502 (2)
C6—H6A	0.9900	C6'—H6'A	0.9900
C6—H6B	0.9900	C6'—H6'B	0.9900
C7—C8	1.383 (2)	C7'—C12'	1.383 (2)
C7—C12	1.390 (2)	C7'—C8'	1.388 (2)
C7—C13	1.5127 (18)	C7'—C13'	1.5125 (18)
C8—C9	1.389 (2)	C8'—C9'	1.385 (2)
C8—H8	0.9500	C8'—H8'	0.9500
C9—C10	1.378 (3)	C9'—C10'	1.383 (3)
C9—H9	0.9500	C9'—H9'	0.9500
C10—C11	1.380 (3)	C10'—C11'	1.375 (3)
C10—H10	0.9500	C10'—H10'	0.9500
C11—C12	1.387 (2)	C11'—C12'	1.389 (2)
C11—H11	0.9500	C11'—H11'	0.9500
C12—H12	0.9500	C12'—H12'	0.9500
C13—O14	1.2436 (17)	C13'—O15'	1.2447 (17)
C13—O15	1.2690 (17)	C13'—O14'	1.2599 (17)
			
C2—N1—H1A	105.8 (9)	C2'—N1'—H1'A	106.7 (11)
C2—N1—H1B	106.8 (9)	C2'—N1'—H1'B	107.8 (10)
H1A—N1—H1B	111.6 (14)	H1'A—N1'—H1'B	110.8 (14)
C2—N1—H1C	111.9 (10)	C2'—N1'—H1'C	111.8 (10)
H1A—N1—H1C	113.4 (14)	H1'A—N1'—H1'C	109.5 (14)
H1B—N1—H1C	107.2 (14)	H1'B—N1'—H1'C	110.2 (15)
N1—C2—C3	111.87 (13)	N1'—C2'—C3'	111.07 (12)
N1—C2—H2A	109.2	N1'—C2'—H2'A	109.4
C3—C2—H2A	109.2	C3'—C2'—H2'A	109.4
N1—C2—H2B	109.2	N1'—C2'—H2'B	109.4
C3—C2—H2B	109.2	C3'—C2'—H2'B	109.4
H2A—C2—H2B	107.9	H2'A—C2'—H2'B	108.0
N4—C3—C2	113.21 (13)	N4'—C3'—C2'	112.22 (13)
N4—C3—H3A	108.9	N4'—C3'—H3'A	109.2
C2—C3—H3A	108.9	C2'—C3'—H3'A	109.2
N4—C3—H3B	108.9	N4'—C3'—H3'B	109.2
C2—C3—H3B	108.9	C2'—C3'—H3'B	109.2
H3A—C3—H3B	107.8	H3'A—C3'—H3'B	107.9
C5—N4—C6	108.42 (11)	C3'—N4'—C6'	110.11 (12)
C5—N4—C3	109.47 (12)	C3'—N4'—C5'	112.79 (12)
C6—N4—C3	111.96 (12)	C6'—N4'—C5'	108.54 (11)
N4—C5—C6^i^	111.48 (13)	N4'—C5'—C6'^ii^	110.65 (12)
N4—C5—H5A	109.3	N4'—C5'—H5'A	109.5
C6^i^—C5—H5A	109.3	C6'^ii^—C5'—H5'A	109.5
N4—C5—H5B	109.3	N4'—C5'—H5'B	109.5
C6^i^—C5—H5B	109.3	C6'^ii^—C5'—H5'B	109.5
H5A—C5—H5B	108.0	H5'A—C5'—H5'B	108.1
N4—C6—C5^i^	111.13 (12)	N4'—C6'—C5'^ii^	111.14 (12)
N4—C6—H6A	109.4	N4'—C6'—H6'A	109.4
C5^i^—C6—H6A	109.4	C5'^ii^—C6'—H6'A	109.4
N4—C6—H6B	109.4	N4'—C6'—H6'B	109.4
C5^i^—C6—H6B	109.4	C5'^ii^—C6'—H6'B	109.4
H6A—C6—H6B	108.0	H6'A—C6'—H6'B	108.0
C8—C7—C12	118.90 (13)	C12'—C7'—C8'	119.15 (12)
C8—C7—C13	119.50 (13)	C12'—C7'—C13'	119.82 (13)
C12—C7—C13	121.57 (12)	C8'—C7'—C13'	120.99 (13)
C7—C8—C9	120.39 (16)	C9'—C8'—C7'	120.50 (14)
C7—C8—H8	119.8	C9'—C8'—H8'	119.7
C9—C8—H8	119.8	C7'—C8'—H8'	119.7
C10—C9—C8	120.31 (16)	C10'—C9'—C8'	119.60 (15)
C10—C9—H9	119.8	C10'—C9'—H9'	120.2
C8—C9—H9	119.8	C8'—C9'—H9'	120.2
C9—C10—C11	119.78 (15)	C11'—C10'—C9'	120.50 (14)
C9—C10—H10	120.1	C11'—C10'—H10'	119.7
C11—C10—H10	120.1	C9'—C10'—H10'	119.7
C10—C11—C12	120.04 (16)	C10'—C11'—C12'	119.65 (16)
C10—C11—H11	120.0	C10'—C11'—H11'	120.2
C12—C11—H11	120.0	C12'—C11'—H11'	120.2
C11—C12—C7	120.57 (14)	C7'—C12'—C11'	120.56 (15)
C11—C12—H12	119.7	C7'—C12'—H12'	119.7
C7—C12—H12	119.7	C11'—C12'—H12'	119.7
O14—C13—O15	123.91 (12)	O15'—C13'—O14'	123.96 (12)
O14—C13—C7	118.44 (12)	O15'—C13'—C7'	118.02 (12)
O15—C13—C7	117.65 (12)	O14'—C13'—C7'	118.01 (12)
			
N1—C2—C3—N4	−66.32 (18)	N1'—C2'—C3'—N4'	−56.03 (18)
C2—C3—N4—C5	164.89 (13)	C2'—C3'—N4'—C6'	168.40 (13)
C2—C3—N4—C6	−74.84 (16)	C2'—C3'—N4'—C5'	−70.19 (16)
C6—N4—C5—C6^i^	57.36 (17)	C3'—N4'—C5'—C6'^ii^	179.90 (11)
C3—N4—C5—C6^i^	179.76 (12)	C6'—N4'—C5'—C6'^ii^	−57.80 (16)
C5—N4—C6—C5^i^	−57.14 (17)	C3'—N4'—C6'—C5'^ii^	−177.99 (12)
C3—N4—C6—C5^i^	−178.01 (11)	C5'—N4'—C6'—C5'^ii^	58.10 (16)
C12—C7—C8—C9	−1.1 (2)	C12'—C7'—C8'—C9'	1.6 (2)
C13—C7—C8—C9	176.94 (13)	C13'—C7'—C8'—C9'	−176.24 (13)
C7—C8—C9—C10	1.0 (2)	C7'—C8'—C9'—C10'	−1.2 (2)
C8—C9—C10—C11	−0.1 (2)	C8'—C9'—C10'—C11'	−0.3 (2)
C9—C10—C11—C12	−0.8 (2)	C9'—C10'—C11'—C12'	1.4 (2)
C10—C11—C12—C7	0.7 (2)	C8'—C7'—C12'—C11'	−0.5 (2)
C8—C7—C12—C11	0.28 (19)	C13'—C7'—C12'—C11'	177.32 (13)
C13—C7—C12—C11	−177.74 (12)	C10'—C11'—C12'—C7'	−0.9 (2)
C8—C7—C13—O14	7.89 (17)	C12'—C7'—C13'—O15'	−6.79 (19)
C12—C7—C13—O14	−174.10 (12)	C8'—C7'—C13'—O15'	171.02 (13)
C8—C7—C13—O15	−172.22 (12)	C12'—C7'—C13'—O14'	172.71 (13)
C12—C7—C13—O15	5.79 (17)	C8'—C7'—C13'—O14'	−9.48 (19)

Symmetry codes: (i) −*x*, −*y*, −*z*; (ii) −*x*, −*y*+2, −*z*+1.

### Hydrogen-bond geometry (Å, º)

**Table:**

*D*—H···*A*	*D*—H	H···*A*	*D*···*A*	*D*—H···*A*
N1—H1*A*···O15^iii^	0.945 (18)	1.833 (19)	2.7739 (17)	173.0 (15)
N1—H1*B*···O15	0.902 (16)	1.895 (17)	2.7836 (15)	167.8 (14)
N1—H1*B*···O14	0.902 (16)	2.632 (16)	3.2580 (16)	127.2 (13)
N1—H1*C*···O14^iv^	0.925 (18)	1.887 (18)	2.7660 (16)	157.9 (14)
N1′—H1′*A*···O14′	0.882 (19)	1.857 (19)	2.7355 (17)	174.2 (15)
N1′—H1′*B*···O14′^v^	0.897 (17)	1.908 (17)	2.7836 (16)	164.8 (15)
N1′—H1′*B*···O15′^v^	0.897 (17)	2.533 (16)	3.1858 (16)	130.1 (13)
N1′—H1′*C*···O15′^vi^	0.916 (18)	1.934 (18)	2.7585 (16)	148.8 (14)

Symmetry codes: (iii) −*x*+1/2, *y*+1/2, −*z*+1/2; (iv) −*x*+1/2, *y*−1/2, −*z*+1/2; (v) −*x*+1/2, *y*+1/2, −*z*+3/2; (vi) *x*, *y*+1, *z*.
